# Clinical impact of tirzepatide on patients with chronic obstructive pulmonary disease

**DOI:** 10.3389/fphar.2026.1838392

**Published:** 2026-05-20

**Authors:** Jheng-Yan Wu, Yu-Min Lin, Wan-Hsuan Hsu, Ting-Hui Liu, Ya-Wen Tsai, Po-Yu Huang, Min-Hsiang Chuang, Chih-Cheng Lai

**Affiliations:** 1 Department of Nutrition, Chi Mei Medical Center, Tainan, Taiwan; 2 Department of Public Health, College of Medicine, National Cheng Kung University, Tainan, Taiwan; 3 Division of Cardiology, Department of Internal Medicine, Chi Mei Hospital, Chiali, Taiwan; 4 Department of Internal Medicine, Chi Mei Medical Center, Tainan, Taiwan; 5 Department of Psychiatry, Chi Mei Medical Center, Tainan, Taiwan; 6 Division of Preventive Medicine, Chi Mei Medical Center, Tainan, Taiwan; 7 Department of Intensive Care Medicine, Chi Mei Medical Center, Tainan, Taiwan; 8 School of Medicine, College of Medicine, National Sun Yat-sen University, Kaohsiung, Taiwan

**Keywords:** COPD, exacerbation, GLP-1RA, mortality, tirzepatide

## Abstract

**Background:**

Chronic obstructive pulmonary disease (COPD) is a prevalent, debilitating condition linked to significant morbidity and mortality. While glucagon-like peptide-1 receptor agonists (GLP-1RAs) have demonstrated clinical benefits for COPD, the effect of tirzepatide on COPD outcomes remains to be fully elucidated.

**Methods:**

This study utilized the TriNetX Global Collaborative Network to analyze data from adults with COPD who were prescribed tirzepatide between 1 January 2022, and 28 February 2025. Patients were divided into two groups: those prescribed tirzepatide and those receiving standard care (control group). Propensity score matching (PSM) was applied to balance covariates. The primary outcome was the risk of COPD exacerbations.

**Results:**

After PSM, the study included 12,110 patients (6,055 in each group). The tirzepatide group was associated with a lower risk of COPD exacerbations (HR, 0.77; 95% CI, 0.63–0.93) compared to the control group. Additionally, tirzepatide use was associated with a lower risk of mortality (HR, 0.52; 95% CI, 0.37–0.73), pneumonia (HR, 0.71; 95% CI, 0.59–0.86), and acute respiratory failure (HR, 0.59; 95% CI, 0.48–0.73).

**Conclusion:**

Tirzepatide use was associated with a potentially reduced risk of COPD exacerbations, lower mortality, and a decreased incidence of pneumonia and acute respiratory failure. These findings should be interpreted with caution, given the inherent limitations of observational study designs, including the possibility of residual confounding and selection bias. The observed associations do not establish causality, and the clinical generalizability of these results remains uncertain. Prospective randomized controlled trials are warranted to confirm these preliminary findings.

## Introduction

Chronic obstructive pulmonary disease (COPD) is characterized by progressive airflow limitation and debilitating symptoms such as dyspnea, cough, and sputum production (Celli and Wedzicha, 2019). It remains a leading cause of global morbidity and mortality, associated with high economic burdens (Chen et al., 2023; Stolz et al., 2022). Exacerbations, acute worsening of respiratory symptoms, accelerate disease progression by increasing lung function decline, systemic inflammation, and dynamic hyperinflation, placing a significant burden on both patients and healthcare systems (Hurst et al., 2020; Bollmeier and Hartmann, 2020). Given these profound impacts, optimizing COPD management and implementing effective strategies to prevent exacerbations are essential for improving patient outcomes and reducing healthcare burdens.

Alongside conventional COPD treatments, emerging evidence suggests glucagon-like peptide-1 receptor agonists (GLP-1RAs), primarily used for type 2 diabetes (T2D) and obesity, may offer therapeutic benefits in respiratory diseases (Lee et al., 2025; Ray et al., 2025). Preclinical studies demonstrate that GLP-1RAs reduce pulmonary inflammation, oxidative stress, and airway remodeling while promoting bronchodilation and surfactant production (Wang et al., 2023; Altintas Dogan et al., 2022). Clinically, a population-based cohort study in the UK disclosed that compared with sulfonylureas, GLP-1 receptor agonists were associated with a 30% decreased risk of severe and moderate exacerbations (Pradhan et al., 2022). In the US, another retrospective, observational, electronic health records-based study revealed that COPD patients with T2D prescribed GLP-1RAs experienced fewer exacerbations compared to those on dipeptidyl peptidase-4 inhibitors (DPP-4i) or sulfonylureas, with parallel declines in severe exacerbation risk (Foer et al., 2023). Similarly, a nationwide cohort study in Taiwan demonstrated that GLP-1RAs could be associated with a lower risk of cardiopulmonary outcomes and all-cause mortality than non-GLP-1RAs in patients with T2D and COPD (Yen et al., 2024). Collectively, these findings suggest that GLP-1RAs represent a promising therapeutic avenue for modulating COPD progression and reducing exacerbation risk, particularly in patients with T2D (Ray et al., 2025; Pradhan et al., 2022; Foer et al., 2023; Yen et al., 2024).

Given these insights, another novel agent, tirzepatide, a dual glucagon-like peptide-1 (GLP-1) and glucose-dependent insulinotropic polypeptide (GIP) receptor agonist, has emerged as a promising alternative with distinct advantages over GLP-1RAs (Druck and er, 2024). While both drug classes improve glycemic control and promote weight loss, tirzepatide has demonstrated superior efficacy (Moiz et al., 2025; Mather et al., 2024; Yao et al., 2024), suggesting that its dual mechanism may offer additional clinical benefits. Beyond its metabolic effects, tirzepatide may also hold relevance for COPD management, given the frequent coexistence of COPD with multiple comorbidities, including cardiovascular disease, metabolic disorders, obesity, and obstructive sleep apnea. These conditions can complicate disease management and worsen prognosis (Fabbri et al., 2023). Notably, tirzepatide has shown beneficial effects on these comorbid conditions, including significant improvements in cardiovascular risk factors, metabolic parameters, and substantial weight reduction (Syed, 2022), which may indirectly benefit COPD progression. However, its potential effects on COPD remain to be fully characterized. In this study, we hypothesize that tirzepatide use is associated with improved outcomes in patients with COPD compared with non-use. To investigate this, we conducted a retrospective study to assess the association between tirzepatide use and clinical outcomes in patients with COPD.

## Methods

### Data sources

This retrospective cohort study was conducted using data obtained from the TriNetX platform, a global federated health research network. The TriNetX Global Collaborative Network provides access to electronic health records, including diagnoses, procedures, medications, laboratory results, and genomic data, from approximately 150 million patients across 146 healthcare organizations (HCOs). The Chi Mei medical center institutional review board approved this TriNetX database study (approval number: 11402-E02). Since the research only used aggregated statistical data from de-identified sources, informed consent requirements were waived. The study adhered to the Strengthening the Reporting of Observational Studies in Epidemiology (STROBE) guidelines. Data access was granted in March 2025, encompassing patient records from 1 January 2022, to 28 February 2025, within the Global Collaborative Network.

### Study design

The study population included adults aged 18 years and older with a documented diagnosis of COPD, identified using the International Classification of Diseases, Tenth Revision, Clinical Modification (ICD-10-CM) codes J41, J42, J43, or J44. Participants were categorized into two groups. The tirzepatide group comprised individuals who initiated tirzepatide therapy following their COPD diagnosis, whereas the control group included individuals who had not received tirzepatide. Patients with a history of tirzepatide use prior to the index date or a documented diagnosis of malignant neoplasms were excluded from the analysis. The index date was defined as the date of the first tirzepatide prescription for the tirzepatide group and the date of COPD diagnosis for the control group. Detailed algorithms used to define patient characteristics, clinical diagnoses, procedures, medications, and laboratory values are provided in Supplementary Table 1.

### Covariates and propensity score matching

After establishing the cohorts, index dates, outcomes, and relevant patient-level covariates that could act as potential confounders, the platform computed covariate values for each individual to generate a covariate matrix. These covariates were obtained within the one-year period preceding the index date. A logistic regression model was then employed to estimate the propensity score, reflecting each patient’s likelihood of belonging to the second cohort based on their baseline characteristics. Using a greedy nearest-neighbor matching algorithm with a caliper of 0.1 pooled standard deviations, patients in the smaller cohort were matched to counterparts in the larger cohort with similar propensity scores. Covariate balance was considered adequate if the standardized mean difference (SMD) was less than 0.1 (Haukoos and Lewis, 2015).

The 1:1 propensity score matching (PSM) included demographic variables such as age, sex, and race; a broad spectrum of comorbidities, including overweight and obesity, malnutrition, alcohol-related disorders, nicotine dependence, hypertension, dyslipidemia, T2D, chronic liver disease, chronic kidney disease, heart failure, atrial fibrillation, ischemic heart disease, pulmonary heart disease, and obstructive sleep apnea; as well as medication use, including antihypertensive and anti-diabetic agents. Laboratory data incorporated into the model included hemoglobin A1c (HbA1c) levels, considered both as a continuous variable and as a categorical variable using a cutoff of >9%. Full definitions and operational codes for all covariates are detailed in Supplementary Table 2.

### Outcomes and follow-up

The primary outcome was acute exacerbation of COPD, while secondary outcomes included all-cause mortality, pneumonia, and acute respiratory failure. Missing data were not imputed. In the survival analysis, patients were censored at the time they ceased to contribute further information. Follow-up for outcomes commenced 1 month after the index date and continued until the occurrence of the outcome event, the last clinical encounter, death, or 1 year post–index date, whichever came first. To reduce the potential for protopathic and ascertainment biases, outcome of interest occurring before the index date were excluded from analysis. Detailed definitions and coding criteria for all outcomes are provided in Supplementary Table 3.

### Statistical analysis

Continuous variables were presented as mean values with corresponding standard deviations (SDs), whereas categorical variables were expressed as frequencies and proportions. For both primary and secondary outcomes, hazard ratios (HRs) and 95% confidence intervals (CIs) were derived using Cox proportional hazards regression models. Survival differences were assessed using Kaplan-Meier curves alongside log-rank tests. All statistical analyses were performed using the TriNetX platform.

### Stratified analysis

Stratified analyses were performed to explore variations in the primary outcome across different categories, including (18–64 and ≥65 years), sex (male and female), status of T2D, obesity, obstructive sleep apnea, and different type of inhaled bronchodilator. Additionally, the analyses explored variations across different inhaled bronchodilator regimens for COPD treatment, including long-acting beta2-agonists (LABA) or long-acting muscarinic antagonists (LAMA), LABA plus LAMA, LABA plus inhaled corticosteroids (ICS), and the combination of LABA, LAMA, and ICS. We further examined the consistency of results by inspecting CI overlap within each stratified group and by calculating interaction p-values to formally assess heterogeneity.

### Additional analysis

To address the potential impact of competing risks associated with the primary outcome, an additional composite outcome comprising both acute exacerbation (AE) and all-cause mortality was analyzed (Hara et al., 2021). Furthermore, recognizing that the effects of tirzepatide may not be immediate, a landmark analysis was conducted to evaluate the primary outcome during follow-up periods beginning at two and three months after the index date, extending up to one year (Morgan, 2019). Additionally, to further clarify the comparative effectiveness of tirzepatide, we performed an active comparator analysis directly comparing tirzepatide with GLP-1RAs. Lastly, E-values were calculated to assess the robustness of the observed associations for both primary and secondary outcomes in the presence of potential unmeasured confounding (VanderWeele and Ding, 2017).

### Control outcome analysis

To explore potential mechanisms underlying the observed associations, we evaluated changes in metabolic parameters, including HbA1c and body mass index (BMI), as positive control outcomes. To assess whether systematic bias was present, we selected the diagnosis of traumatic brain injury and skin cancer as negative control outcomes (Arnold and Ercumen, 2016).

## Results

### Patients’ selection

**FIGURE 1:**
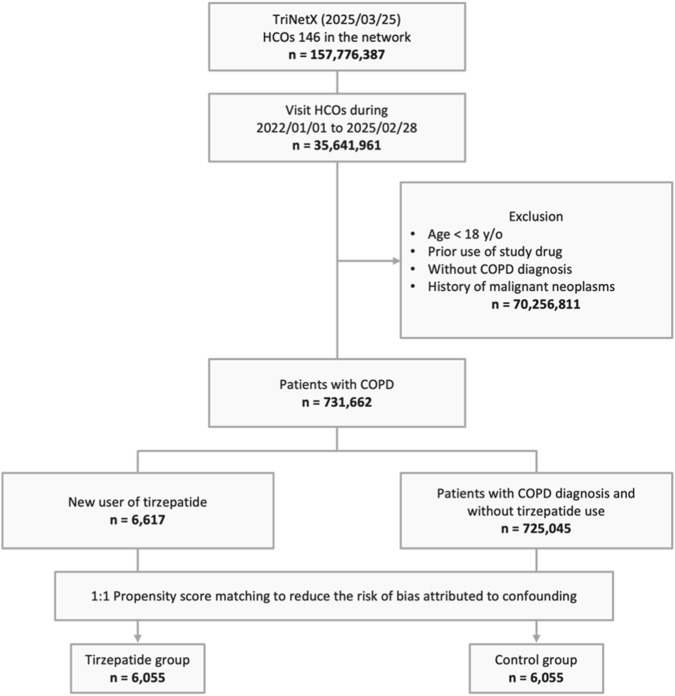
Study cohort process. COPD, chronic obstructive pulmonary disease; HCOs, healthcare organizations; y/o, years old.

An initial cohort of 157,776,387 individuals was identified through a screening of 146 HCOs within the Global Collaborative Network of the TriNetX platform on 25 March 2025. Among them, 35,641,961 individuals had more than two visits to HCOs between 1 January 2022, and 28 February 2025. After applying exclusion criteria, 70,256,811 individuals were removed, resulting in a final cohort of 731,662 adults diagnosed with COPD. Within this population, 6,617 individuals were identified as new users of tirzepatide, while 725,045 who did not receive tirzepatide served as the control group. PSM was then performed, yielding 6,055 well-matched patients in both the tirzepatide and control groups (Figure 1).

### Demographic features of included patients

Before PSM, patients in the tirzepatide group (n = 6,617) were younger, with a mean age of 62.4 ± 10.6 years, compared to those in the control group (n = 725,045), who had a mean age of 65.3 ± 13.6 years. The proportion of female patients was higher in the tirzepatide group (62.1%) than in the control group (50.3%). Racial distribution also differed significantly, with a greater percentage of White individuals in the tirzepatide group (73.4% vs. 56.5%). Additionally, more patients among tirzepatide users had HbA1c levels ≥9% compared to the control group (1.2%). Comorbidities were notably more prevalent in the tirzepatide group, particularly overweight and obesity (48.1% vs. 6.0%), nicotine dependence (16.2% vs. 7.9%), hypertension (59.7% vs. 21.2%), T2D (45.5% vs. 10.2%), hyperlipidemia (57.5% vs. 15.9%), chronic liver disease (10.7% vs. 2.7%), chronic kidney disease (17.2% vs. 5.5%), cerebrovascular disease (7.3% vs. 4.2%), heart failure (19.1% vs. 5.8%), atrial fibrillation/flutter (12.1% vs. 5.1%), ischemic heart disease (26.9% vs. 9.9%), pulmonary heart disease (8% vs. 2.1%), and obstructive sleep apnea (30.3% vs. 3.3%). Regarding medication use, a substantially higher percentage of tirzepatide users had prior exposure to antihypertensive including angiotensin-converting enzyme inhibitor, angiotensin receptor block, beta-blocker, calcium channel blocker, diuretics and anti-diabetic drugs, including biguanide, sulfonylurea, thiazolidinedione, dipeptidyl peptidase-4 inhibitor, sodium-glucose cotransporter-2 inhibitor, and glucagon-like peptide-1 receptor agonist. Following PSM, both groups consisted of 6,055 patients with well-balanced baseline characteristics (all SMD <0.1) (Table 1).

**TABLE 1:** Baseline characteristics of patients before and after matching.

Variables	Before matching			After matching		
	Tirzepatide group (n = 6,617)	Control group (n = 725,045)	SMD	Tirzepatide group (n = 6,055)	Control group (n = 6,055)	SMD
Age at index, years						
Mean ± SD	62.4 ± 10.6	65.3 ± 13.6	0.235	62.4 ± 10.6	62.3 ± 11.5	0.009
Sex, n (%)						
Female	4,110 (62.1)	360,180 (50.3)	0.24	3,739 (61.8)	3,776 (62.4)	0.013
Male	2,495 (37.7)	354,737 (49.5)	0.24	2,304 (38.1)	2,254 (37.2)	0.017
Race, n (%)						
White	4,860 (73.4)	404,717 (56.5)	0.361	4,416 (72.9)	4,414 (72.9)	0.001
Black or african american	1,113 (16.8)	84,033 (11.7)	0.146	1,034 (17.1)	1,157 (19.1)	0.053
Asian	149 (2.3)	37,360 (5.2)	0.157	146 (2.4)	109 (1.8)	0.043
Other race	139 (2.1)	13,103 (1.8)	0.02	131 (2.2)	124 (2)	0.008
Unknown race	222 (3.4)	169,109 (23.6)	0.621	210 (3.5)	155 (2.6)	0.053
HbA1c, %						
≥9, n (%)	664 (10.6)	8,234 (1.2)	0.407	583 (10.1)	643 (11.1)	0.034
Comorbidities, n (%)						
Overweight and obesity	3,181 (48.1)	42,955 (6)	1.076	2,835 (46.8)	2,891 (47.7)	0.019
Malnutrition	41 (0.6)	5,549 (0.8)	0.019	36 (0.6)	34 (0.6)	0.004
Alcohol related disorders	132 (2)	10,142 (1.4)	0.045	102 (1.7)	77 (1.3)	0.034
Nicotine dependence	1,072 (16.2)	56,428 (7.9)	0.258	898 (14.8)	891 (14.7)	0.003
Hypertension	3,948 (59.7)	152,133 (21.2)	0.851	3,538 (58.4)	3,670 (60.6)	0.044
Dyslipidemia	3,805 (57.5)	113,843 (15.9)	0.957	3,435 (56.7)	3,520 (58.1)	0.028
Type 2 diabetes	3,916 (45.5)	94,321 (10.2)	0.857	3,480 (44.7)	3,803 (48.9)	0.083
Chronic liver disease	705 (10.7)	19,476 (2.7)	0.322	633 (10.5)	620 (10.2)	0.007
Chronic kidney disease	1,139 (17.2)	39,252 (5.5)	0.376	1,011 (16.7)	1,022 (16.9)	0.005
Cerebrovascular diseases	484 (7.3)	30,206 (4.2)	0.133	418 (6.9)	424 (7)	0.004
Heart failure	1,263 (19.1)	41,873 (5.8)	0.409	1,062 (17.5)	1,057 (17.5)	0.002
Atrial fibrillation and flutter	802 (12.1)	36,407 (5.1)	0.253	707 (11.7)	721 (11.9)	0.007
Ischemic heart diseases	1,778 (26.9)	71,138 (9.9)	0.448	1,557 (25.7)	1,600 (26.4)	0.016
Pulmonary heart disease	532 (8)	15,333 (2.1)	0.271	450 (7.4)	411 (6.8)	0.025
Obstructive sleep apnea	2,006 (30.3)	23,533 (3.3)	0.775	1,761 (29.1)	1,694 (28)	0.025
Antihypertensives, n (%)						
Angiotensin-converting enzyme inhibitor	1,376 (17.9)	62,627 (8.1)	0.294	1,215 (17.4)	1,314 (18.8)	0.037
Angiotensin receptor blocker	2,088 (27.2)	62,490 (8.1)	0.517	1,853 (26.6)	2,008 (28.8)	0.050
Beta blockers	2,680 (34.9)	115,007 (14.9)	0.475	2,364 (33.9)	2,574 (36.9)	0.063
Calcium channel blockers	1,925 (25.1)	87,929 (11.4)	0.360	1,680 (24.1)	1,891 (27.1)	0.069
Diuretics	3,023 (39.4)	109,004 (14.1)	0.595	2,620 (37.5)	2,873 (41.2)	0.074
Anti-diabetic drugs, n (%)						
Biguanide	2,102 (27.4)	35,520 (4.6)	0.654	1,863 (26.7)	2,016 (28.9)	0.049
Sulfonylurea	676 (8.8)	14,349 (1.9)	0.313	610 (8.7)	687 (9.8)	0.038
Thiazolidinedione	200 (2.61)	2,835 (0.4)	0.186	180 (2.6)	208 (3)	0.024
Dipeptidyl peptidase-4 inhibitor	320 (4.2)	10,673 (1.4)	0.170	291 (4.2)	320 (4.6)	0.020
Sodium-glucose cotransporter-2 inhibitor	1,460 (19.0)	10,756 (1.4)	0.609	1,276 (18.3)	1,215 (17.4)	0.023
Glucagon-like peptide-1 receptor agonists	2,533 (38.3)	7,738 (1.1)	1.059	2,322 (38.3)	2,171 (35.9)	0.052

Standardized mean difference (SMD) < 0.1 is considered a small difference.

### Primary outcome

**FIGURE 2:**
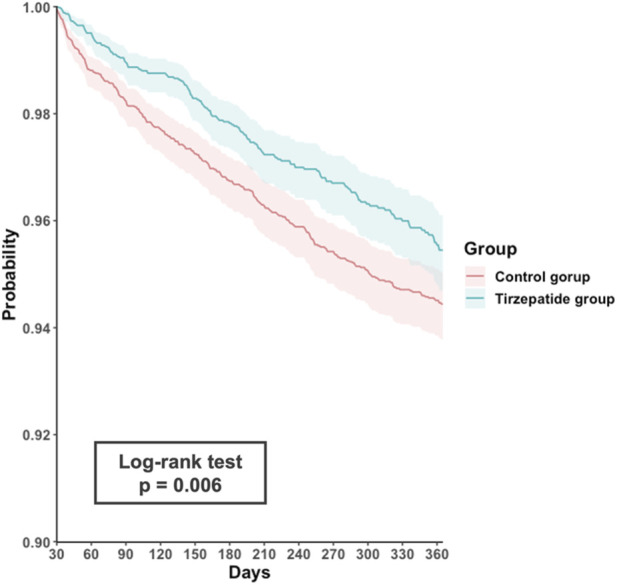
Kaplan-Meier time-to-event free curves of the acute exacerbation comparison to tirzepatide and control groups.

During the follow-up period, 166 episodes of COPD exacerbation occurred in the tirzepatide group, with an incidence rate of 3.9 per 100 person-years, compared to 285 events in the control group, which had an incidence rate of 4.7 per 100 person-years. The risk of COPD exacerbation was significantly lower in the tirzepatide group (HR, 0.77; 95% CI, 0.63–0.93; p = 0.006). The Schoenfeld residuals test confirmed that the proportional hazards assumption was not violated (p > 0.05), and an E-value of 1.9 suggested robust findings against potential unmeasured confounders. Kaplan-Meier survival analysis indicated a significantly lower cumulative incidence of COPD exacerbations in the tirzepatide group compared to the control group (log-rank test, p = 0.006, Figure 2).

**FIGURE 3:**
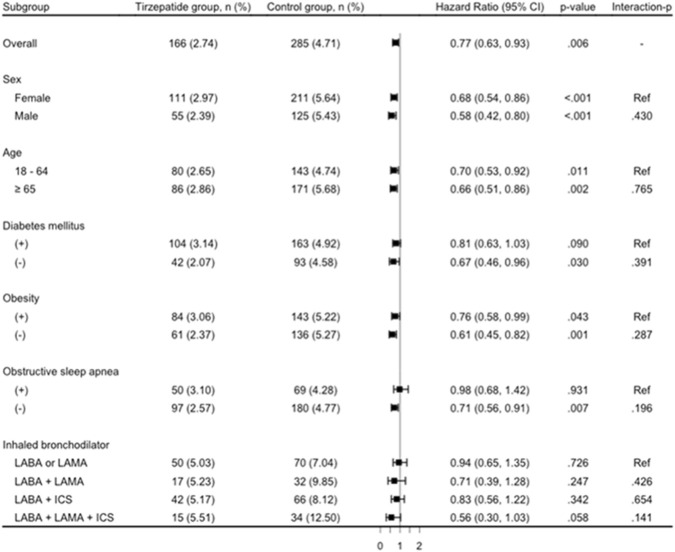
Primary outcome within subgroup analyses for the matched tirzepatide and control groups. CI, confidence interval; ICS, inhaled corticosteroids; LABA, long-acting beta2-agonist; LAMA, long-acting muscarinic antagonist.

Stratified analyses demonstrated consistent findings with HR < 1 across all subgroups (Figure 3). Sex-based analysis showed that females had a lower risk (HR, 0.68; 95% CI, 0.54–0.86), as did males (HR, 0.58; 95% CI, 0.42–0.80). In the age-stratified analysis, the HR was 0.70 (95% CI, 0.53–0.92) for individuals aged 18–64 years and 0.66 (95% CI, 0.51–0.86) for those aged ≥65 years. Among patients with T2D, the risk reduction was not statistically significant (HR, 0.81; 95% CI, 0.63–1.03), whereas those without diabetes showed a significant benefit (HR, 0.67; 95% CI, 0.46–0.96). Similarly, obesity-stratified analysis indicated a lower risk in both obese (HR, 0.76; 95% CI, 0.58–0.99) and non-obese individuals (HR, 0.61; 95% CI, 0.45–0.82). For patients with OSA, no significant association was observed (HR, 0.98; 95% CI, 0.68–1.42), whereas those without OSA benefited from tirzepatide (HR, 0.71; 95% CI, 0.56–0.91). Analysis based on the use of inhaled bronchodilators and ICS showed that tirzepatide was associated with a lower risk of COPD exacerbation in patients using LABA or LAMA (HR, 0.94; 95% CI, 0.65–1.35), LABA plus LAMA (HR, 0.71; 95% CI, 0.39–1.28), LABA plus ICS (HR, 0.83; 95% CI, 0.56–1.22), and the triple therapy of LABA, LAMA, and ICS (HR, 0.56; 95% CI, 0.30–1.03). However, none of these associations reached statistical significance. Lastly, no statistically significant interaction was observed across any of the examined subgroups (all interaction p > 0.05).

### Secondary outcomes

Similarly, the risk of all-cause mortality alone was lower in the tirzepatide group, with 45 events (incidence rate: 1.0 per 100 person-years) compared to 119 events (incidence rate: 1.9 per 100 person-years) in the control group (HR, 0.52; 95% CI, 0.37–0.73; p < 0.001). In addition, the incidence of pneumonia was also lower in the tirzepatide group (166 events, 3.9 per 100 person-years) compared to the control group (306 events, 5.1 per 100 person-years), with an HR of 0.71 (95% CI, 0.59–0.86; p < 0.001). Lastly, the risk of acute respiratory failure was significantly reduced in the tirzepatide group (HR, 0.59; 95% CI, 0.48–0.73; p < 0.001), with 125 events (incidence rate: 2.9 per 100 person-years) compared to 277 events (incidence rate: 4.8 per 100 person-years) in the control group. All proportional hazard assumptions were met (Schoenfeld test p > 0.05), and the observed E-values were 3.3, 2.2, and 2.8 for all-cause mortality, pneumonia, and acute respiratory failure respectively, suggesting strong resistance to potential unmeasured confounding (Table 2).

**TABLE 2:** The hazard ratios for both the primary and secondary outcomes comparing the matched tirzepatide group with the control group.

Outcome	Tirzepatide group (n = 6,055)		Control group (n = 6,055)		HR (95% CI)	*P* value	E-value (95% LCL)
	Events (n)	Incidence rate per 100 person-years	Events (n)	Incidence rate per 100 person-years			
Primary outcome							
Acute exacerbation	166	3.9	285	4.7	0.77 (0.63,0.93)	0.006	1.9 (1.4)
Secondary outcomes							
All-cause mortality	45	1.0	119	1.9	0.52 (0.37,0.73)	<0.001	3.3 (2.1)
Pneumonia	166	3.9	306	5.1	0.71 (0.59,0.86)	<0.001	2.2 (1.6)
Acute respiratory failure	125	2.9	277	4.8	0.59 (0.48,0.73)	<0.001	2.8 (2.1)

CI, confidence interval; HR, hazard ratio; ICH, intracranial hemorrhage; LCL, lower confidence limit.

### Additional and sensitivity tests

For the composite outcome of acute exacerbation and all-cause mortality, 205 events occurred in the tirzepatide group (incidence rate: 4.8 per 100 person-years) compared to 395 events in the control group (incidence rate: 6.5 per 100 person-years), resulting in a significantly lower risk in the tirzepatide group (HR, 0.69; 95% CI, 0.58–0.81; p < 0.001) (Supplementary Table 4).

A landmark analysis of outcomes was conducted over two time periods: 2-month to 1-year and 3-month to 1-year. During these time frames, tirzepatide remained associated with a lower risk of COPD exacerbation (Supplementary Table 5). In the active comparator analysis, the comparison between tirzepatide and GLP-1RAs yielded consistent results, with tirzepatide demonstrating a lower risk of primary and secondary outcomes (Supplementary Table 6). For positive control outcomes, tirzepatide was associated with greater reductions in HbA1c and BMI compared with both the control and GLP-1RA groups (Supplementary Table 7). In the negative control outcome analysis, no significant differences were found in the hazard of traumatic brain injury and skin cancer between the tirzepatide and control groups (Supplementary Table 8).

## Discussion

This study represents the first large-scale investigation involving over 12,000 patients, demonstrating that tirzepatide use is associated with a significantly lower risk of COPD exacerbations compared to non-users. Our findings indicate a 23% reduction in exacerbation risk, with consistent results across subgroups stratified by age, sex, and comorbidity. Kaplan-Meier analysis further supports these results, showing a reduced cumulative incidence of COPD exacerbations in the tirzepatide group. The clinical implications of these findings are profound. COPD exacerbations are a major driver of disease progression, hospitalizations, and mortality, and frequent episodes can accelerate lung function decline while increasing the risk of cardiovascular events, reduced quality of life, and long-term morbidity (Hurst et al., 2020; Calabria et al., 2024; Celli et al., 2021; Alqahtani et al., 2020). Preventing such exacerbations remains a critical therapeutic goal in COPD management, yet effective pharmacologic options have been limited (Viniol and Vogelmeier, 2018). Our study suggests that tirzepatide may represent a promising therapeutic strategy, offering potential benefits that extend well beyond its established metabolic effects and potentially transforming our approach to COPD treatment.

In addition to its effect on COPD exacerbations, this study comprehensively examined the association between tirzepatide use and mortality. Our findings revealed a significant 48% reduction in all-cause mortality among tirzepatide users, a result with potential implications. This survival benefit was accompanied by substantial reductions in acute respiratory failure (41%) and pneumonia (29%), underscoring tirzepatide’s broader impact on respiratory health outcomes. Currently, triple therapy combining a LABA/LAMA/ICS represents the only pharmacologic approach demonstrated to improve survival in COPD (Rabe et al., 2020; Lipson et al., 2020; Lai et al., 2022). Our finding that tirzepatide may confer an additional survival benefit is particularly noteworthy, given the limited pharmacologic options for reducing mortality in this challenging disease. These promising results demand rigorous further investigation to fully characterize tirzepatide’s therapeutic potential and its implications for COPD management.

In this study, we conducted a stratified analysis based on the presence of underlying health conditions. Our analyses consistently showed that tirzepatide was associated with a lower risk of COPD exacerbation across subgroups of patients with T2D, obesity, or obstructive sleep apnea. Although these differences did not reach statistical significance, likely due to limited sample sizes, they remain clinically meaningful. To further explore whether these subgroup findings reflect true effect modification, we performed interaction tests for diabetes status and OSA status. None of the interaction p-values reached statistical significance, suggesting no robust evidence for heterogeneity of treatment effect. Therefore, the observed significant associations in non-diabetic and non-OSA strata but not in their counterparts are likely due to random variability around a consistent direction of effect rather than true subgroup-specific differences.

COPD is frequently complicated by coexisting comorbidities, which not only increase the symptom burden but also contribute to higher rates of exacerbations, hospitalizations, and mortality (Spece et al., 2018; Eroglu et al., 2019; Chatila et al., 2008; Skajaa et al., 2023). Taken together, our findings suggest a promising therapeutic potential for tirzepatide in managing COPD patients with concurrent health conditions. However, further large-scale studies are warranted to validate these findings.

While our study does not elucidate the exact mechanisms underlying the beneficial effects of tirzepatide on COPD outcomes, several plausible explanations exist. Preclinical studies suggest that GLP-1 receptor activation confers direct pulmonary benefits by reducing inflammation through downregulation of key proinflammatory cytokines, such as interleukin-6 and monocyte chemoattractant protein-1, while simultaneously promoting bronchodilation and enhancing surfactant production (Wang et al., 2023; Altintas Dogan et al., 2022). Clinically, tirzepatide achieves superior metabolic improvements, including greater weight loss and better glycemic control compared to other antidiabetic agents (Yao et al., 2024; Ayesh et al., 2024). These metabolic optimizations may alleviate respiratory burden by reducing diaphragmatic strain and improving oxygen utilization (Mafort et al., 2016; De Soomer et al., 2024). Moreover, retrospective studies consistently support the potential of incretin-based therapies in improving COPD outcomes (Ray et al., 2025; Pradhan et al., 2022; Foer et al., 2023; Yen et al., 2024). Finally, given that cardiovascular events are well-established triggers of COPD exacerbations, the additional cardiovascular benefits conferred by tirzepatide may further contribute to the observed reduction in exacerbation risk. Together, these factors may partially explain the favorable outcomes observed in our study.

The major strength of this study lies in its large-scale, real-world design. To our knowledge, this is the first population-based investigation to demonstrate a significant association between tirzepatide use and a reduced risk of COPD exacerbations and mortality. The comprehensive dataset allowed for rigorous subgroup analyses, which consistently showed benefits across diverse patient populations, thereby enhancing the generalizability of our findings. We also examined a wide range of clinically meaningful outcomes, including acute respiratory failure and pneumonia, further supporting the potential respiratory benefits of tirzepatide. By leveraging real-world data from multiple healthcare organizations, we ensured both external validity and direct relevance to clinical practice. Additionally, the application of multiple analytic methods, such as propensity score matching and sensitivity analyses, further strengthened the robustness of our results.

However, this study has several limitations. First, its retrospective observational design precludes establishing causal relationships, and prospective randomized controlled trials are needed to validate our findings. Second, the use of electronic health records as the primary data source may introduce heterogeneity and bias. Variability in coding practices, potential misclassification of diagnoses or outcomes, and incomplete capture of clinical variables could have affected the accuracy of our analyses. Moreover, missing baseline data were not imputed. Because TriNetX aggregates data from electronic medical records collected during routine clinical care, completeness cannot be guaranteed, and missing values remain unaltered. If the missingness was not completely at random, this could introduce bias and affect the reliability of the results. Future studies should strive to minimize missing data and consider statistical approaches such as multiple imputation to strengthen the robustness of the findings. Third, despite propensity score matching, some unmeasured factors such as socioeconomic status, access to healthcare, medication adherence, or environmental exposures were not available in the database and may have contributed to residual confounding. Nevertheless, the high E-value observed in this study indicates that the potential influence of these residual factors is likely limited. Fourth, the differing definitions of the index date between groups—the date of first tirzepatide prescription for the tirzepatide group versus the date of COPD diagnosis for the control group—may introduce immortal time bias. Specifically, patients in the tirzepatide group must have survived and remained event-free from the time of COPD diagnosis until tirzepatide initiation, a period during which they were not yet classified as treated. This gap creates a window of artificially “immortal” time that is not equivalently present in the control group, potentially inflating the apparent benefits of tirzepatide. Although our landmark analyses beginning at two and three months post-index date yielded consistent results, this bias cannot be fully eliminated with the current design, and future studies employing active comparator designs or time-varying exposure definitions would be better positioned to address this issue. Fifth, the follow-up period was limited to one year, which may be insufficient to fully assess the long-term impact of tirzepatide on COPD progression, exacerbation patterns, or the development of chronic complications. Sixth, COPD severity was not directly measured, as spirometric parameters and GOLD staging were unavailable in the TriNetX database. Instead, we relied on medication use (inhaled bronchodilators with or without inhaled corticosteroids) as a proxy for disease severity. While this approach allowed for some adjustment, it is an indirect and imperfect measure, and the lack of direct lung function data limits the interpretation of our findings. Seventh, the severity of COPD exacerbations could not be assessed, which restricts our ability to determine whether tirzepatide influences not only the frequency but also the clinical burden of exacerbations. Eighth, the specific clinical indications for tirzepatide prescriptions were not available in the TriNetX database. While many patients were likely prescribed tirzepatide for obesity or T2D, the relatively low prevalence of these conditions in our cohort suggests that some patients may have received it for other metabolic disorders, weight management, or prediabetes. If tirzepatide was used primarily for weight reduction or glycemic control, the observed benefits on COPD outcomes may have been mediated largely through improvements in metabolic status rather than direct pulmonary effects. This possibility should be considered when interpreting the findings. Ninth, information on tirzepatide dose, treatment duration, and medication adherence was not available within the TriNetX platform, as these data are not systematically recorded in routine electronic health records. The inability to account for dosing intensity or adherence limits our capacity to establish dose–response relationships, assess whether the magnitude of benefit varies with treatment exposure, or determine whether non-adherence attenuated the observed effects. Tenth, the TriNetX platform does not systematically capture adverse events associated with tirzepatide use. Clinically relevant side effects, such as nausea, vomiting, diarrhea, or treatment discontinuation due to drug intolerance, are not reliably coded in routine clinical care data, precluding a comprehensive safety assessment of tirzepatide in this COPD population. Eleventh, the substantial reduction in sample size after propensity score matching, particularly in certain subgroup analyses, may limit the generalizability of our findings to the broader COPD population. Finally, mortality may act as a competing risk for COPD exacerbations. To address this, we performed an additional composite outcome analysis that included both exacerbations and mortality, and the beneficial association with tirzepatide persisted. Nonetheless, the potential for competing risks underscores the complexity of outcome interpretation in COPD populations with high comorbidity burdens. Taken together, these limitations highlight the preliminary nature of our findings. Future prospective, randomized controlled trials with longer follow-up and more comprehensive clinical data are warranted to confirm the association, elucidate the underlying mechanisms, and clarify whether tirzepatide’s potential protective effects in COPD are mediated through metabolic improvements, direct pulmonary actions, or both.

## Conclusion

This study provides compelling evidence that tirzepatide may be associated with a reduced risk of exacerbations and improved survival outcomes in patients with COPD. These findings highlight tirzepatide’s potential as a significant adjuvant therapeutic option in COPD management, particularly in mitigating exacerbation frequency and enhancing overall patient survival. However, while these results are encouraging, further prospective randomized controlled trials are essential to establish causal relationships and comprehensively explore the long-term effects of tirzepatide on respiratory health outcomes.

## Data Availability

The original contributions presented in the study are included in the article/Supplementary Material, further inquiries can be directed to the corresponding author.
