# Supplementary material for: Clinical impact of tirzepatide on patients with chronic obstructive pulmonary disease
Source: Front Pharmacol. 2026 May 20;17:1838392. doi: 10.3389/fphar.2026.1838392 (PMC13231402; doi:10.3389/fphar.2026.1838392)
Supplement: Supplementary file 1 [file Supplementaryfile1.docx]

**eTable 1.** Demographic, diagnostic, procedural, medication, visit, and laboratory codes used in the definition of the cohorts

| **Category** | **Code** | **Description** |
| --- | --- | --- |
| **Tirzepatide group** | | |
| **#1**: At least 18 years old | | |
| Demographics | Age | Age (at least 18 years) |
| **#2**: Patients with COPD treated with tirzepatide.  (# 2.1 must be fulfilled after #2.2) | | |
| **#2.1:** Patients with tirzepatide | | |
| Medication | RxNorm:2601723 | Tirzepatide |
| **#2.2**: Patients with COPD | | |
| Diagnosis | UMLS:ICD10CM:J41 | Simple and mucopurulent chronic bronchitis |
| Diagnosis | UMLS:ICD10CM:J42 | Unspecified chronic bronchitis |
| Diagnosis | UMLS:ICD10CM:J43 | Emphysema |
| Diagnosis | UMLS:ICD10CM:J44 | Other chronic obstructive pulmonary disease |
| **#4**: Visit HCOs since 2022 | | |
| Visit | Visit | Visit since 2022 |
| **#5**: Cannot have history of malignant neoplasms | | |
| Diagnosis | UMLS:ICD10CM:C81-C96 | Malignant neoplasms of lymphoid, hematopoietic and related tissue |
| Diagnosis | UMLS:ICD10CM:C76-C80 | Malignant neoplasms of illdefined, other secondary and unspecified sites |
| Diagnosis | UMLS:ICD10CM:C30-C39 | Malignant neoplasms of respiratory and intrathoracic organs |
| Diagnosis | UMLS:ICD10CM:C15-C26 | Malignant neoplasms of digestive organs |
| **Control group** | | |
| **#1**: At least 18 years old | | |
| Demographics | Age | Age (at least 18 years) |
| **#2**: Patients with COPD | | |
| Diagnosis | UMLS:ICD10CM:J41 | Simple and mucopurulent chronic bronchitis |
| Diagnosis | UMLS:ICD10CM:J42 | Unspecified chronic bronchitis |
| Diagnosis | UMLS:ICD10CM:J43 | Emphysema |
| Diagnosis | UMLS:ICD10CM:J44 | Other chronic obstructive pulmonary disease |
| **#3**: **#3**: Without tirzepatide | | |
| Medication | RxNorm:2601723 | Tirzepatide |
| **#4**: Visit HCOs since 2022 | | |
| Visit | Visit | Visit since 2022 |
| **#5**: Cannot have history of malignant neoplasms | | |
| Diagnosis | UMLS:ICD10CM:C81-C96 | Malignant neoplasms of lymphoid, hematopoietic and related tissue |
| Diagnosis | UMLS:ICD10CM:C76-C80 | Malignant neoplasms of illdefined, other secondary and unspecified sites |
| Diagnosis | UMLS:ICD10CM:C30-C39 | Malignant neoplasms of respiratory and intrathoracic organs |
| Diagnosis | UMLS:ICD10CM:C15-C26 | Malignant neoplasms of digestive organs |

**eTable 2.** Demographic, diagnostic, and laboratory codes used in the definition of covariates

| **Category** | **Code** | **Description** |
| --- | --- | --- |
| Demographics | AI | Age at index |
| Demographics | F | Female |
| Demographics | 2106-3 | White |
| Demographics | UNK | Unknown Race |
| Demographics | 2054-5 | Black or African American |
| Demographics | 2028-9 | Asian |
| Demographics | 2131-1 | Other Race |
| Diagnosis | F10 | Alcohol related disorders |
| Diagnosis | F17 | Nicotine dependence |
| Diagnosis | E11 | Type 2 diabetes mellitus |
| Diagnosis | E78 | Disorders of lipoprotein metabolism and other lipidemia |
| Diagnosis | I10 | Essential (primary) hypertension |
| Diagnosis | N18 | Chronic kidney disease (CKD) |
| Diagnosis | I50 | Heart failure |
| Diagnosis | I20-I25 | Ischemic heart disease |
| Diagnosis | I48 | Atrial fibrillation |
| Diagnosis | I60-I69 | Cerebrovascular disease |
| Diagnosis | K70-K77 | Liver disease |
| Diagnosis | I26-I28 | Pulmonary heart disease and diseases of pulmonary circulation |
| Diagnosis | G47.33 | Obstructive sleep apnea |
| Medication | A10BA | Biguanides |
| Medication | A10BB | Sulfonylureas |
| Medication | A10BF | Alpha glucosidase inhibitors |
| Medication | A10BG | Thiazolidinediones |
| Medication | A10BH | Dipeptidyl peptidase 4 (DPP-4) inhibitors |
| Medication | A10BK | Sodium-glucose co-transporter 2 (SGLT2) inhibitors |
| Medication | A10BJ | Glucagon-like peptide-1 (GLP-1) analogues |
| Medication | CV100 | BETA BLOCKING AGENTS |
| Medication | CV200 | CALCIUM CHANNEL BLOCKERS |
| Medication | CV700 | DIURETICS |
| Medication | CV800 | Ace inhibitors |
| Medication | CV805 | Angiotensin ii inhibitor |
| Lab | 9037 | HbA1c |

**eTable 3.** Diagnostic, visit, and procedural codes used in the definition of outcomes

| **Category** | **Code** | **Description** |
| --- | --- | --- |
| **#1**: Acute exacerbation | | |
| Diagnosis | UMLS:ICD10CM:J44.1 | Chronic obstructive pulmonary disease with acute exacerbation |
| **#2**: All-cause mortality (have any of the following) | | |
| Visit | Deceased | Deceased |
| Diagnosis | UMLS:ICD10CM:R99 | Ill-defined and unknown cause of mortality |
| **#3**: Pneumonia | | |
| Diagnosis | UMLS:ICD10CM:J18 | Pneumonia |
| **#4**: Acute respiratory failure | | |
| Diagnosis | UMLS:ICD10CM:J96.0 | Acute respiratory failure |

**eTable 4.** Cox proportional hazards analysis of composite outcome including acute exacerbation and all-cause mortality

| Outcome | No. of patients with outcome | | HR (95% CI) | *P* value |
| --- | --- | --- | --- | --- |
|  | Tirzepatide group  (n = 6,055) | Control group  (n = 6,055) |  |  |
| Acute exacerbation and all-cause mortality | 205 | 395 | 0.69 (0.58,0.81) | < .001 |

**eTable 5**. Sensitivity analysis with Landmark analysis of primary outcome

| Outcome | HR (95% CI) | *P* value |
| --- | --- | --- |
|  |  |  |
| 2-months to 1-year |  |  |
| Acute exacerbation | 0.75 (0.63,0.90) | 0.002 |
| 3-months to 1-year |  |  |
| Acute exacerbation | 0.78 (0.65,0.93) | 0.007 |

**eTable 6.** The hazard ratios for both the primary and secondary outcomes comparing the matched tirzepatide group with the GLP-1RA group.

| Outcome | Tirzepatide group (n = 28,015) | | GLP-1RA group (n = 28,015) | | HR (95% CI) | *P* value | E-value (95% LCL) |
| --- | --- | --- | --- | --- | --- | --- | --- |
|  | Events (n) | Incidence rate  per 100 person-years | Events (n) | Incidence rate  per 100 person-years |  |  |  |
| Primary outcome |  |  |  |  |  |  |  |
| Acute exacerbation | 1,998 | 8.7 | 2,700 | 9.6 | 0.93 (0.88,0.99) | 0.017 | 1.4 (1.1) |
| Secondary outcomes |  |  |  |  |  |  |  |
| All-cause mortality | 254 | 1.1 | 495 | 1.8 | 0.68 (0.58,0.79) | <.001 | 1.1 (1.8) |
| Pneumonia | 1,043 | 4.5 | 1,507 | 5.4 | 0.89 (0.82,0.96) | 0.004 | 1.5 (1.3) |
| Acute respiratory failure | 816 | 3.5 | 1,167 | 4.2 | 0.90 (0.82,0.98) | 0.016 | 1.5 (1.2) |

**eTable 7**. Comparison of HbA1c and body mass index changes among the tirzepatide and comparator groups.

| Outcomes | Tirzepatide group | Comparator group | P value for difference |
| --- | --- | --- | --- |
| Tirzepatide vs. control groups (n = 6,055 in each group) | | | |
| HbA1c (%), Mean ± SD | -0.4 ± 2.3 | 0.5 ± 2.2 | < 0.001 |
| BMI (kg/m^2^), Mean ± SD | -1.9 ± 11.7 | 3.7 ± 24.4 | < 0.001 |
| Tirzepatide vs. GLP-1RA groups (n = 28,015 in each group) | | | |
| HbA1c (%), Mean ± SD | -0.5 ± 2.3 | -0.4 ± 2.6 | < 0.001 |
| BMI (kg/m^2^), Mean ± SD | -1.8 ± 11.7 | 0.1 ± 11.8 | < 0.001 |

P value was calculated based on the change in between-group HbA1c and BMI difference from baseline.

**eTable 8.** Negative outcomes between the tirzepatide group and the control group

| Outcome | HR (95% CI) | *P* value |
| --- | --- | --- |
|  |  |  |
| Traumatic brain injury | 0.66 (0.29,1.54) | 0.337 |
| Skin cancer | 1.13 (0.74,1.73) | 0.575 |
